# Pathology of A(H5N8) (Clade 2.3.4.4) Virus in Experimentally Infected Chickens and Mice

**DOI:** 10.1155/2019/4124865

**Published:** 2019-07-04

**Authors:** Elena A. Prokopyeva, Vsevolod A. Zinserling, You-Chan Bae, Yongkuk Kwon, Olga G. Kurskaya, Ivan A. Sobolev, Peter M. Kozhin, Andrey Komissarov, Artem Fadeev, Vladimir Petrov, Alexander M. Shestopalov, Kirill A. Sharshov

**Affiliations:** ^1^Department of Experimental Research, Federal Research Center for Basic and Translational Medicine, Novosibirsk 630117, Russia; ^2^Medical Department, Novosibirsk State University, Novosibirsk 630090, Russia; ^3^Institute of Experimental Medicine, Almazov National Federal Research Centre, Saint Petersburg 197341, Russia; ^4^Avian Disease Division, Animal and Plant Quarantine Agency, Gimcheon-si 39660, Republic of Korea; ^5^Department of etiology and epidemiology, Smorodintsev Research Institute of Influenza, Saint Petersburg 197376, Russia

## Abstract

The emergence of novel highly pathogenic avian influenza viruses (HPAIVs) in migratory birds raises serious concerns as these viruses have the potential to spread during fall migration. We report the identification of novel HPAIV A(H5N8) clade 2.3.4.4 virus that was isolated from sick domestic duck at commercial farm during the second wave of spread that began in October and affected poultry (ducks; chiсkens) in several European regions of Russia and Western Siberia in 2016. The strain was highly lethal in experimental infection of chickens and mice with IVPI = 2.34 and MLD_50_ = 1.3log_10_⁡ EID_50_, accordingly. Inoculation of chickens with the HPAIV A/H5N8 demonstrated neuroinvasiveness, multiorgan failure, and death of chickens on the 3^rd^ day post inoculation. Virus replicated in all collected organ samples in high viral titers with the highest titer in the brain (6.75±0.1 log_10_TCID_50_/ml). Effective virus replication was found in the following cells: neurons and glial cells of a brain; alveolar cells and macrophages of lungs; epithelial cells of a small intestine; hepatocytes and Kupffer cells of a liver; macrophages and endothelial cells of a spleen; and the tubular epithelial cells of kidneys. These findings advance our understanding of histopathological effect of A(H5N8) HPAIV infection.

## 1. Introduction

Different strains of influenza viruses play an important role in human and animal pathology. A(H5N1) highly pathogenic avian influenza viruses have caused considerable economic damage to the global poultry industry and pose a serious threat to public health. About 20 years ago, A/goose/Guangdong/1/1996(H5N1) (briefly Gs/Gd/96), clade 2.3.4 precursor of currently circulating H5N1 HPAIVs, was first isolated in farmed geese at Sanshui, Foshan, a rural area in southern China [1]. The H5N1 HPAIVs did not disappear and later variants spread further to the Mideast, Europe, and Africa. The diversity of influenza viruses and viral transmission between domestic poultry and wild birds might have resulted in the appearance of A(H5N8) clade 2.3.4.4 Gs/GD-lineage HPAIV, which first emerged during poultry outbreak in China in 2010 [2], and in domestic ducks and migratory birds in South Korea in 2014 [3]. The extensive distribution of c HPAIVs during last two outbreaks formed 2 distinct groups of A(H5N8) viruses: group A (Buan-like) and group B (Gochang-like). Group A viruses predominated in South Korea [3] and were subsequently isolated in northeast Siberia in September 2014 [4]. In 2016, two waves of virus dissemination in Russia were reported: the first wave began in June in Western Siberia and affected aquatic birds, while the second wave began in October and affected poultry (ducks; chickens) in several European regions of Russia and Western Siberia [5, 6]. To date, many isolates of A(H5N8) have been identified in North America, Africa, and Europe and continue to cause outbreaks among wild birds and poultry [7–9]. Pathologic lesions in HPAIV-infected birds are extremely variable and depend on many factors, including virus strain, host species, age and immune status, and natural environment. However, the detailed pathobiology of A(H5N8) Gs/GD-lineage HPAIV such as host adaptation, tissue tropism, histopathologic lesions, infectivity, and transmissibility remains unclear. Here we study the infectivity and pathogenicity of A(H5N8) clade (2.3.4.4) HPAIVs from the Siberia, Russia, in chickens.

## 2. Materials and Methods

### 2.1. Virus

A/domestic duck/Siberia/49feather/2016(H5N8) (A/49feather/2016), clade 2.3.4.4, was isolated in October 2016 from the feathers of the sick domestic duck at commercial farm in Siberia. The virus was propagated in 10-days old embryonated chicken eggs and stored at -70°C. The 50% egg infectious dose (EID_50_) and the 50% tissue culture infectious dose (TCID_50_) for MDCK cells were determined as described previously [10].

### 2.2. Animal Experiments

Animal experiments were approved by the Ethics Committee of the Federal Research Center of Fundamental and Translational Medicine (No. 2017-15).

#### 2.2.1. Experimental Infection of Chickens

The intravenous pathogenicity index (IVPI) test for A/49feather/2016 was performed as described in the OIE Manual [11]. Ten 6-week-old specific-pathogen-free (SPF) chickens were intravenously (iv) inoculated with 0.1 ml of 1:10 diluted infective allantoic fluid (containing 10^6.0^  EID_50_ of the virus). The chickens were examined daily for clinical signs of the disease for 10 days. The pathogenicity index was calculated as the mean score per bird per observation.

Brain, lungs, liver, kidneys, intestine, heart, and spleen samples were collected from three birds immediately after death that happened on the third day post inoculation (dpi). A full set of organs were collected from each bird and fixed in 10% neutral buffered formalin, paraffin-embedded, sectioned, and stained with hematoxylin-and-eosin for histopathologic evaluation. Duplicate sections were stained by immunohistochemical (IHC) methods to determine influenza viral antigen distribution. Briefly, sections were stained with a mouse monoclonal antibody against influenza A virus nucleoprotein (MCA-400, AbD Serotec, Duesseldorf, Germany), followed by a biotinylated goat anti-mouse IgG secondary antibody. Bound antibodies were detected with an avidin-biotin detection system (Ventana Medical Systems, Tucson, AZ). The RedMap kit (Ventana Medical Systems) served as substrate chromogen. Another duplicate section of brain, lungs, heart, kidney, liver, and spleen were stained by immunofluorescence (IF). The slides were deparaffinized and rehydrated. Heat-induced epitope retrieval was performed using a microwave and 10 mM sodium citrate buffer (pH 6.0) with 0.05% Tween 20. Slides were washed 2 times for 5 minutes in PBS with 0.025% Triton X-100, washed with PBS, and placed for 40 minutes into blocking buffer containing 1% BSA in PBS. Preparations were incubated for 1 hour at RT with primary mouse antibodies against influenza A NP (AA5H, Netherlands), then washed 3 times for 5 minutes in PBS, and incubated for 1h at RT with secondary antibodies to mouse immunoglobulins conjugated with fluorochrome AlexaFluor488 (Abcam, ab150105). Slides were washed 3 times for 5 minutes in PBS. Cell nuclei were contrasted with 5 *μ*M 4,6-diamino-2phenyl indole (DAPI), briefly washed with deionized water, and mounted in the FluoroShield mounting medium. Images were captured under a laser scanning confocal microscope LSM710/NLO (Carl Zeiss, Germany).

Portions of lungs, brain, intestine, liver, spleen, and kidneys were also collected and homogenized in 1 mL PBS and centrifuged; the supernatant was collected and virus was titrated in MDCK cells from initial dilution of 1:10. Virus titers were calculated by Kerber technique with Ashmarin-Vorobyov modification [12] according to the following formula: log_10_⁡TCID50/ml = lgDn- *δ*(ΣLi – 0.5) where

  Dn is maximum effect of dose;
  Li is the ratio of the number of wells with cytopathic effect to the total number of wells infected with this dose;
  i is number of dose;
 
*δ* is the logarithm of virus dilutions.

#### 2.2.2. Experimental Infection of Mice

The 50% mouse lethal dose (MLD_50_) of A/49feather/2016 was determined for 6-week-old female BALB/c mice (Federal Budgetary Research Institution State Research Center of Virology and Biotechnology VECTOR, Novosibirsk, Russia). Groups of 10 mice were lightly anesthetized with diethyl ether (2-4 % in the inhaled mixture) and intranasally inoculated with 50 *μ*l of the phosphate buffer saline (PBS) containing 10^0^–10^7^EID_50_ of the virus. Mice were observed daily for death for 14 days after inoculation. MLD_50_ was then calculated by Kerber method with Ashmarin-Vorobyov modification as described previously.

### 2.3. Sequencing and Phylogenetic Analysis

Full‐genome amplification of A/49feather/2016 virus was performed according to Zhou et al. [13]. Nextera XT sample preparation was used to obtain libraries for next‐generation sequencing; full‐length genome sequences were obtained using Illumina MiSeq. Sequences of completed genome were submitted to Epiflu GISAID database (www.gisaid.org/). GISAID BLAST was used to analyze the identity of strain A/49feather/2016. Multiply alignment and identity percent calculation were performed by MEGA 7 [14].

### 2.4. Determination of Susceptibility to Neuraminidase Inhibitors

The susceptibility of strain A/49feather/2016 to oseltamivir (Hoffmann-La Roche, Basel, Switzerland) was evaluated by neuraminidase (NA) inhibition assays as previously reported [14]. Briefly, virus was standardized to a NA activity level 10-fold higher than that of the background, as measured by the production of a fluorescent product from methylumbelliferyl-N-acetylneuraminic acid (MUNANA) substrate (Sigma-Aldrich, Darmstadt, Germany). Drug susceptibility profiles were determined by the extent of NA inhibition after incubation with 3-fold serial dilutions of NAIs. The 50% inhibitory concentrations (IC_50_) were determined from the dose-response curve.

This work involved the use of equipment from the Multi-Access Center “Modern Optical Systems” of the Federal Research Center of Fundamental and Translational Medicine (Novosibirsk, Russia).

All methods were performed in accordance with the relevant guidelines and regulations.

## 3. Results and Discussion

In 2016, A(H5N8) virus spread widely in Russia within two waves. The first summer wave affected wild birds (great crested grebe) in Tuva (Siberia), and the second wave in autumn affected poultry (gadwall, duck, chicken) in several regions of European Russia (Kurgan, Kalmykia) and Western Siberia [5, 6]. GISAID and GenBank nucleotide sequences analyzed in 2016-2017 suggest A(H5N8) influenza virus was widespread and infected both wild and domestic birds, therefore posing the risk of transmission to mammals and humans. No human cases of A(H5N8) virus are reported. However, its prevalence and multiple outbreaks among wild and domestic birds globally make it a serious public health concern.

During earlier monitoring in Western Siberia we isolated low pathogenic avian influenza viruses from wild duck feathers, which suggest the viruses can easily spread among birds during grooming [15]. It is therefore important to assess the pathogenic potential and distribution of viruses isolated during the second wave of spread in Russia.

Here we report the data for A/domestic duck/Siberia/49feather/2016(H5N8) (A/49feather/2016) that was isolated in October 2016 from feathers of sick domestic duck. A/49feather/2016 virus is phylogenetically similar to A(H5Nx) viruses (mainly A(H5N8)) isolated in Russia during 2016-2017 such as A(H5N8) strains A/great crested grebe/Uvs-Nuur Lake/341/2016, A/common tern/Uvs-Nuur Lake/26/2016, A/gray heron/Uvs-Nuur Lake/20/2016, A/wild_duck/Tatarstan/3059/2016, and A(H5N2) strain isolated in Kostroma (A/chicken/Kostroma/1717/2017_PA). In 2016-2017 A(H5N8) strains genetically closely related to A/49feather/2016 were also isolated in many countries of Eurasia and Africa (see Table 1). The degree of identity between coding sequences (coding region) of most genome segments (heterotrimeric polymerase complex with PB1 and PA subunits, nucleoprotein (NP), neuraminidase (NA), matrix protein (MP), and nonstructural protein (NS)) was higher than that of the Russian strains, which may indicate several independent introductions of the virus to Russia.

### 3.1. Virus Replication in Experimentally Inoculated Chickens and Mice

**Figure 1:**
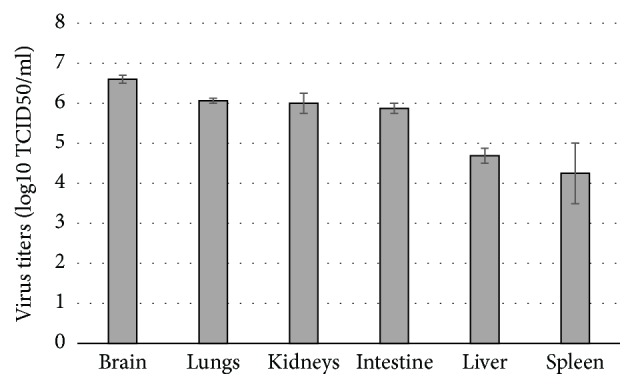
Mean titers of A/domestic duck/Siberia/49feather/2016(H5N8) virus in organs of chickens. Chickens were infected iv with 10^6^  EID_50_/100 *μ*l of A/domestic duck/Siberia/49feather/2016(H5N8) virus. Note: viral titers were determined in MDCK cell culture by Kerber method and expressed as log_10_⁡   TCID_50_ in 1 ml of studied sample as М±CI95, where M is an arithmetic mean value and CI is a confidence interval.

Intravenous (iv) inoculation of three 6-week-old chickens with 10^6.0^ 50% egg infectious dose (EID_50_) of A/49feather/2016 virus led to 100% mortality (IVPI = 2.34) within 3 days. In all organ samples collected on the third day post inoculation (dpi), virus replicated in high viral titers with the highest titer in brain (6.75±0.1 log10TCID_50_/ml) (see Figure 1).

The 50% mouse lethal dose (MLD_50_) was determined by intranasal inoculation with serial dilutions of the virus. A/49feather/2016 was highly pathogenic for mice, with an MLD_50_ of 1.3log_10_EID_50_. All infected mice developed symptoms including body weight loss, ruffled fur, hunched posture, and shivering.

### 3.2. Phenotypic Assay

Oseltamivir susceptibility data were analyzed by standard method [16]. Interpretation of IC_50_ values was based on the World Health Organization Influenza Antiviral Working Group (WHO-AVWG) criteria established for influenza A virus [17]: for normal inhibition (NI) (influenza A <10-fold above normal inhibition), for reduced inhibition (RI) (influenza A 10- to 100-fold above normal inhibition), and for highly reduced inhibition (HRI) (influenza A >100-fold above normal inhibition). Here we compared A/49feather/2016 with vaccine strain А/California/07/2009(H1N1)pdm09 that was isolated in pandemic period and demonstrated normal inhibition by oseltamivir. The comparison confirmed NI of A/49feather/2016.

### 3.3. Microscopic Lesions, Viral Antigen Distribution, and Viral Load in Chicken Organs

**Figure 2:**
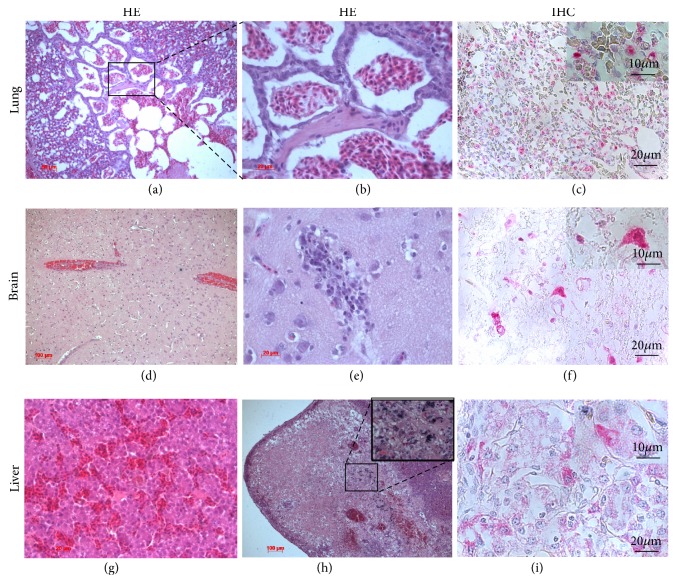
Histological lesions and immunohistochemical detection of viral antigen in lungs, brain, and liver of chicken infected with A(H5N8) HPAI virus; viral antigen staining in red. Note: (a) erythrocytes in the parabronchial lumen and atria of the lung. Bar = 50 *μ*m. (b) The same. Bar = 20 *μ*m. (c) Viral antigen in the alveolar macrophages and alveolar cells. Bar = 20 *μ*m. (d) Venous and capillary hyperemia of the brain. Bar = 100 *μ*m. (e) Local area of brain infiltration. Bar = 20 *μ*m. (f) Many AIV antigens are found in neurons (insert with magnification x1000) and glial cells. Bar = 20 *μ*m. (g) The venous and capillary hyperemia in liver. Bar = 20 *μ*m. (h) The dystrophic changes at a vast area of liver parenchyma; the predominance of active macrophages; and a large number of mast cells, basophils, and eosinophils. Bar = 100 *μ*m. (i) Viral antigen in the Kupffer cell (insert with magnification x1000) and in hepatocytes (bar = 20 *μ*m). Insert demonstrates viral antigen presence in hepatocyte. Magnification x1000. HE: hematoxylin-and-eosin staining; IHC: immunohistochemical staining.

**Figure 3:**
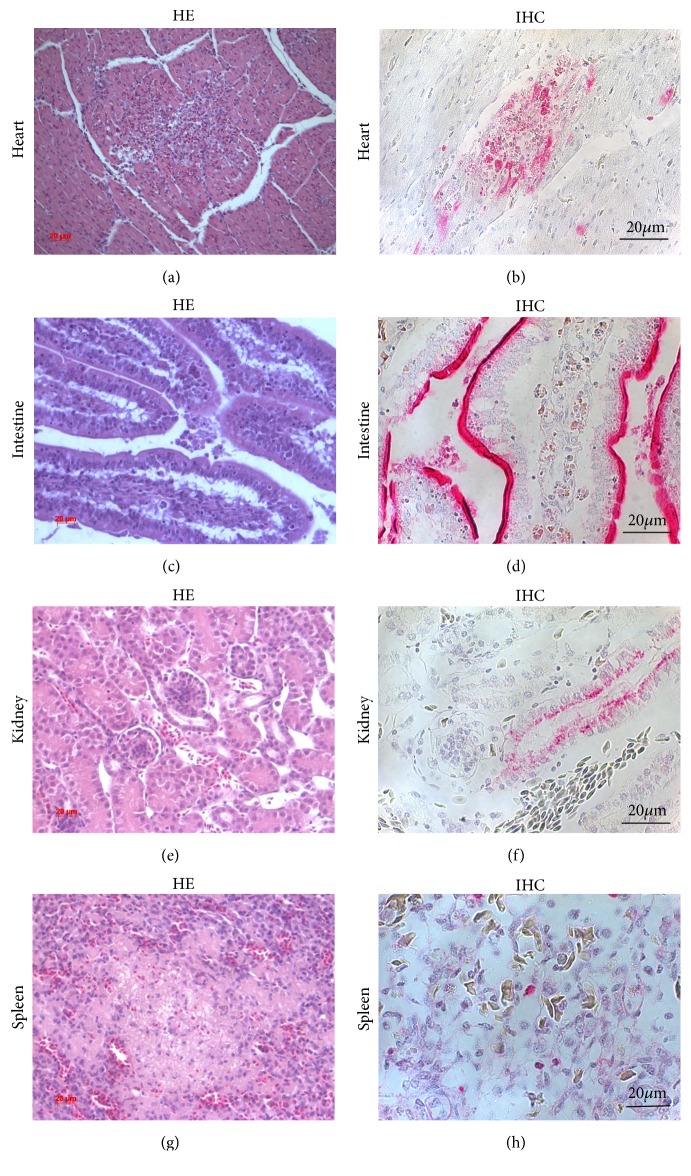
Histological lesions and immunohistochemical detection of viral antigen in heart, intestine, kidneys, and spleen of chicken infected with HPAI A(H5N8) virus; viral antigen staining in red. Note: (a) the lymphohistiocytic myocarditis. Bar = 20 *μ*m. (b) Viral antigen in myocytes. Bar = 20 *μ*m. (c) Intensive mononuclear infiltration of the small intestine; desquamation of epithelium. Bar = 20 *μ*m. (d) Viral antigen in epithelial cells. Bar = 20 *μ*m. E Venous and capillary hyperemia of kidney. Bar = 20 *μ*m. (f) Viral antigens in kidney tubular epithelial cells. Magnification x40. (g) Local area of necrosis in spleen. Bar = 20 *μ*m. (h) Viral antigen in spleen macrophages. Bar = 20 *μ*m. HE: hematoxylin-and-eosin staining; IHC: immunohistochemical staining.

To study microscopic lesions and sites of virus replication, we made a comparative analysis of organs from infected chickens at 3 dpi and from 2 uninfected chickens (controls). Hematoxylin-and-eosin (HE), IHC, and IF staining for AI virus nucleoprotein antigen were performed (see Figures 2 and 3, Suppl. mat.).

#### 3.3.1. Lungs

Atriums of lungs alveoli and airbags containing serous exudate and macrophages were decreased. Considerable amount of nucleated erythrocytes (hemorrhages) was seen in parabronchial lumens, atriums, and air capillaries. Ciliated and goblet cells of respiratory (pseudostratified) epithelium were partly proliferating and partly underwent alterative changes (dystrophy; hyperproduction of mucus). Endothelial cells of blood vessels were swollen and partly destroyed. We also identified perivascular edema, hemorrhages, and edema with a high protein content (3/3) (see Figures 2(a) and 2(b)). Viral antigen staining was observed in alveolar cells, alveolar macrophages (3/3), and viral titers were 6.06±0.06 log_10_TCID_50_/ml (see Figure 2(c) and magnification). These changes indicate severe lesions leading to acute respiratory insufficiency.

#### 3.3.2. Brain

Mild focal malacia was identified in all chickens. Perivascular cuffs consisting of lymphocytes were presented predominantly in cerebral cortex. Veins and capillaries were congested. Edema with a high protein content was present (3/3) (see Figures 2(d) and 2(e)). Viral antigen staining was observed in neurons and glial cells (see Figure 2(f) and magnification), and viral titers were 6.75±0.1 log_10_TCID_50_/ml. Thus, vasculitis and viral-induced lesions of neurons and glial cells were observed, which is related to the neuroinvasiveness of HPAI A(H5N8) virus.

The neuroinvasiveness of A(H5N8) HPAIV was shown in earlier studies [14, 18, 19] and caused lesions similar to those induced by H5N2, H5N6, and H5N8 viruses in domestic Pekin ducks [20], by HPAIV A(H5N8) in fattening ducks [14], and by A(H5N1) in mice [21]. Viral titers in the brains of H5N6- and H5N8-infected ducks were significantly higher than those of H5N2-infected ducks [20].

Mild focal malacia, necrosis of the neurons, and disorders of blood rheology in all examined cases indicated a high neurotropism of the A(H5N8) HPAIV, which was confirmed by positive virologic results and by IHC and IF analyses. Virus dissemination to brain leading to severe neurological dysfunction is considered one of the causes of high influenza A(H5N8) virulence in chickens.

#### 3.3.3. Liver

We observed venous and hepatic sinuses hyperemia. Mild congestion and thrombosis were also seen (1/3). Local areas of leukocyte infiltration primarily near the large blood vessels were identified (3/3) (see Figure 2(g)). Local area of inflammation in liver parenchyma with degradation of hepatocytes with a predominance of macrophages and numerous mast cells, basophils, and eosinophils was detected (1/3) (see Figure 2(h) and magnification). Viral antigen staining was observed in hepatocytes and Kupffer cells, and viral titers were 4.69±0.28 log_10_TCID_50_/ml (see Figure 2(i) and magnification). Thus, liver had disturbances of blood supply and focal hepatitis.

#### 3.3.4. Heart

Multifocal lymphohistiocytic myocarditis and degradation of cardiac myocytes were observed (see Figure 3(a)). The accumulation of myocardial interstitial oedema fluid was identified (3/3). Viral antigen staining was observed in myocytes (see Figure 3(b)).

#### 3.3.5. Intestine

Desquamation of epithelial layer was observed in villi. Intestinal epithelium displayed hyperplasia of goblet cells. Slight mononuclear infiltration in submucosa was identified the. Focal areas of necrosis were detected in mucosal lamina propria (3/3) (see Figure 3(c)). Viral antigen staining was present in epithelial cells of villi (see Figure 3(d)), and viral titers were 5.75±0,13 log_10_TCID_50_/ml. Thus, catarrhal-desquamative enteritis with high level of viral antigen in inflammatory cells was observed in a small intestine.

#### 3.3.6. Kidneys

Foci of acute necrosis and hemorrhage caused by virus were observed in kidneys (1/3). Viral antigen staining was present in epithelial cells of proximal tubule of nephron, and viral titers were 6.0±0.35 log_10_TCID_50_/ml (see Figures 3(e) and 3(f)).

#### 3.3.7. Spleen

Venous and capillary congestion was observed. Mononuclear infiltration was present primarily near the large veins. Multifocal necrosis was seen (3/3) (see Figure 3(g)). Viral antigen was present in macrophages and endothelial cells, and viral titers were 4.25±1.06 log_10_TCID_50_/ml (see Figure 3(h)).

In general, IF staining was more intensive in brain, lungs, and heart. NP viral antigen staining was negligible in kidneys and spleen (not shown). The presence of the viral antigen detected by IF was in agreement with the IHC analysis and histopathological changes (see Suppl. Mat.).

In this study we investigated influenza A(H5N8) virus, isolated during second wave of spread in 2016 in Western Siberia. It showed highly virulent features (IVPI = 2.34); multiorgan dissemination (brain, heart, intestine, liver, spleen, and kidneys) in inoculated chickens. Virus effectively replicated in all collected organs in high viral titers, and the highest titer was in the brain (6.75±0.07 log_10_TCID_50_/ml). A/49feather/2016 showed high pathogenicity in mice (MLD_50_ = 1.3log_10_EID_50_), which may indicate the ability of the virus to infect mammal hosts, and has the potential to cause high mortality in chickens. The identified amino acid changes require further study to reveal their role in highly lethal infection, which may indicate the ability of the virus to infect mammal hosts. Virus was effectively inhibited by oseltamivir and showed inhibition within normal IC_50_ susceptibility range.

Histopathology of avian influenza caused by different strains has been described by several authors [11, 22–24], who demonstrated the ability of the virus to multiplicate in different organs and cell types causing necrotic lesions. However, inflammatory reactions varied significantly in different experiments and birds. Thus, an appropriate histopathological characteristic is vital for every novel strain.

The spillover of A(H5N8) HPAIV to domestic poultry poses a serious threat to animal health sector, particularly in Siberia that experienced A(H5N1) outbreak in 2005 [25]. Strict biosecurity measures are therefore needed to protect poultry farms from viral entry.

## 4. Conclusions

Novel highly pathogenic avian influenza virus A(H5N8) (clade 2.3.4.4) was isolated in October 2016 from feathers of a sick domestic duck at commercial farm in Siberia. This pathogen was highly lethal in experimental infection of chickens and mice with IVPI = 2.34 and MLD_50_ = 1.3log_10_⁡EID_50_, accordingly. We identified the structural elements of chickens internal organs damaged by HPAIV A(H5N8):

- in lungs, alveolar macrophages and alveolar cells;
- in brain, neurons and glial cells;
- in liver, hepatocytes and Kupffer cells;
- in heart, myocytes;
- in intestine, epithelial cells;
- in kidneys, epithelial cells of proximal tubule of nephron;
- in spleen, macrophages and endothelial cells.

This information can be useful to study HPAIV infection caused by A(H5N8) virus that continues to circulate.

## Figures and Tables

**Table 1:** Identity table.

	Russian		Non Russian	
	Strain	% of identity	Strain	% of identity
PB2	A/great_crested_grebe/Uvs-Nuur_Lake/341/2016 (H5N8)	99.65	A/green-winged_teal/Egypt/877/2016 (H5N8)	99.87

PB1	A/great_crested_grebe/Uvs-Nuur_Lake/341/2016 (H5N8)	99.78	A/Bar-headed_Goose/Qinghai/BTY18-LU/2016 (H5N8)	99.65

PA	A/chicken/Kostroma/1717/2017 (H5N2)	99.67	A/painted_stork/India/10CA03/2016 (H5N8)	99.86

HA	A/wild_duck/Tatarstan/3059/2016 (H5N8)	99.47	A/mallard_duck/Korea/WA137/2017 (H5N8)	99.41

NP	A/mallard/Chany/313/2016 (H1N1)	98.00	A/green-winged_teal/Egypt/877/2016 (H5N8)	99.93

NA	A/black-headed_gull/Tyva/41/2016 (H5N8)	99.65	A/chicken/Korea/H903/2017 (H5N8)	99.79

MP	A/chicken/Kostroma/1717/2017 (H5N2)	99.69	A/green-winged_teal/Egypt/871/2016 (H5N8)	100.00
			A/mallard_duck/Korea/WA137/2017 (H5N8)	

NS	A/black-headed_gull/Tyva/41/2016 (H5N8)	99.52	A/painted_stork/India/10CA03/2016 (H5N8)	100.00

## Data Availability

The data used to support the findings of this study are available from the corresponding author upon request.

## Supplementary Materials

Supplementary Materials Immunofluorescence detection of viral antigen in inner organs of the chicken infected with HPAI A(H5N8) virus. Note: a: brain, b: lungs, and c: heart. Green: intracellular influenza A NP protein stained with anti-influenza A NP antibody (AA5H, Netherlands); blue: nuclei stained with 4′,6-diamidino-2-phenylindole (DAPI). Images were acquired by means of the LSM710/NLO microscope, using Plan-Apochromat 63x objective (Carl Zeiss, Germany).

## References

[1] Wan X. F. (1998). *Isolation and Characterization of Avian Influenza Viruses in China*.

[2] Zhao K., Gu M., Zhong L. (2013). Characterization of three H5N5 and one H5N8 highly pathogenic avian influenza viruses in China. *Veterinary Microbiology*.

[3] Jeong J., Kang H., Lee E. (2014). Highly pathogenic avian influenza virus (H5N8) in domestic poultry and its relationship with migratory birds in South Korea during 2014. *Veterinary Microbiology*.

[4] Marchenko V. Y., Susloparov I. M., Kolosova N. P. (2015). Influenza A(H5N8) virus isolation in Russia, 2014. *Archives of Virology*.

[5] Lee D., Sharshov K., Swayne D. E. (2017). Novel reassortant clade 2.3.4.4 avian influenza A(H5N8) virus in wild aquatic birds, russia, 2016. *Emerging Infectious Diseases*.

[6] Marchenko V. Y., Susloparov I. M., Komissarov A. B. (2017). Reintroduction of highly pathogenic avian influenza A/H5N8 virus of clade 2.3.4.4. in Russia. *Archives of Virology*.

[7] Venkatesh D., Poen M. J., Bestebroer T. M. (2018). Avian influenza viruses in wild birds: virus evolution in a multihost ecosystem. *Journal of Virology*.

[8] Napp S., Majó N., Sánchez-Gónzalez R., Vergara-Alert J. (2018). Emergence and spread of highly pathogenic avian influenza A(H5N8) in Europe in 2016-2017. *Transboundary and Emerging Diseases*.

[9] Yehia N., Naguib M. M., Li R. (2018). Multiple introductions of reassorted highly pathogenic avian influenza viruses (H5N8) clade 2.3.4.4b causing outbreaks in wild birds and poultry in Egypt. *Infection, Genetics and Evolution*.

[10] The World Organisation for Animal Health (2004). *(OIE) Manual of Diagnostic Tests and Vaccines for Terrestrial Animals (Mammals, Birds and Bees)*.

[11] Mo I. P., Brugh M., Fletcher O. J., Rowland G. N., Swayne D. E. (1997). Comparative pathology of chickens experimentally inoculated with avian influenza viruses of low and high pathogenicity. *Avian Diseases*.

[12] Ashmarin I., Vorobyov A. (1962). *Statisticheskie Metody V Mikrobiologicheskich Issledovanijach*.

[13] Zhou B., Donnelly M. E., Scholes D. T. (2009). Single‐reaction genomic amplification accelerates sequencing and vaccine production for classical and Swine origin human influenza A viruses. *Journal of Virology*.

[14] Kumar S., Stecher G., Tamura K. (2016). MEGA7: molecular evolutionary genetics analysis version 7.0 for bigger datasets. *Molecular Biology and Evolution*.

[15] De Marco M. A., Delogu M., Sivay M. (2014). Virological evaluation of avian influenza virus persistence in natural and anthropic ecosystems of western siberia (novosibirsk region, summer 2012). *PLoS ONE*.

[16] World Health Organization (2011). *Global Influenza Surveillance Network. Manual for the Laboratory Diagnosis and Virological Surveillance of Influenza*.

[17] World Health Organization Meetings of the WHO working group on surveillance of influenza antiviral susceptibility—Geneva.

[18] Kim H., Kwon Y., Jang I. (2015). Pathologic changes in wild birds infected with highly pathogenic avian influenza A(H5N8) viruses, south korea, 2014. *Emerging Infectious Diseases*.

[19] Anis A., AboElkhair M., Ibrahim M. (2018). Characterization of highly pathogenic avian influenza H5N8 virus from Egyptian domestic waterfowl in 2017. *Avian Pathology*.

[20] Sun H., Pu J., Hu J. (2016). Characterization of clade 2.3.4.4 highly pathogenic H5 avian influenza viruses in ducks and chickens. *Veterinary Microbiology*.

[21] Sharshov K., Prokopyeva E., Susloparov I. (2015). Neuropathological effect of clade 2.3.2 H5N1 influenza virus isolated from wild birds. *Journal of Emerging Diseases and Virology*.

[22] Pantin-Jackwood M. J., Costa-Hurtado M., Bertran K., DeJesus E., Smith D., Swayne D. E. (2017). Infectivity, transmission and pathogenicity of H5 highly pathogenic avian influenza clade 2.3.4.4 (H5N8 and H5N2) United States index viruses in Pekin ducks and Chinese geese. *Veterinary Research*.

[23] Nakatani H., Nakamura K., Yamamoto Y., Yamada M., Yamamoto Y. (2005). Epidemiology, pathology, and immunohistochemistry of layer hens naturally affected with H5N1 highly pathogenic avian influenza in japan. *Avian Diseases*.

[24] Stoute S., Chin R., Crossley B. (2016). Highly pathogenic eurasian H5N8 avian influenza outbreaks in two commercial poultry flocks in california. *Avian Diseases*.

[25] Sharshov K., Romanovskaya A., Uzhachenko R. (2010). Genetic and biological characterization of avian influenza H5N1 viruses isolated from wild birds and poultry in Western Siberia. *Archives of Virology*.

