# Supplementary figures and images for: Pathology of A(H5N8) (Clade 2.3.4.4) Virus in Experimentally Infected Chickens and Mice
Source: Interdiscip Perspect Infect Dis. 2019 Jul 4;2019:4124865. doi: 10.1155/2019/4124865 (PMC6637675; doi:10.1155/2019/4124865)

Brain

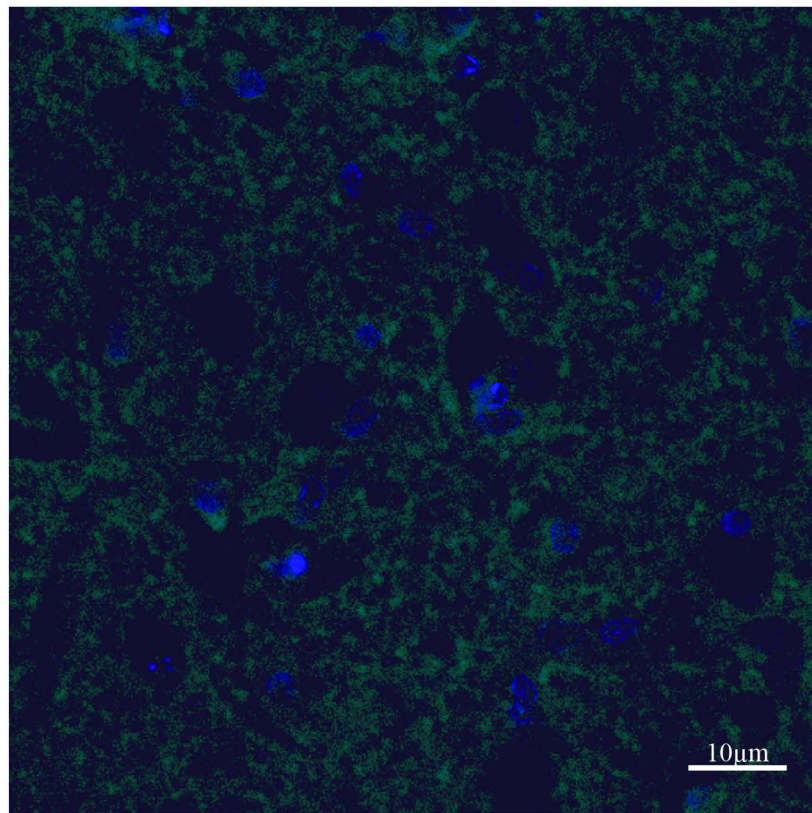

Lung

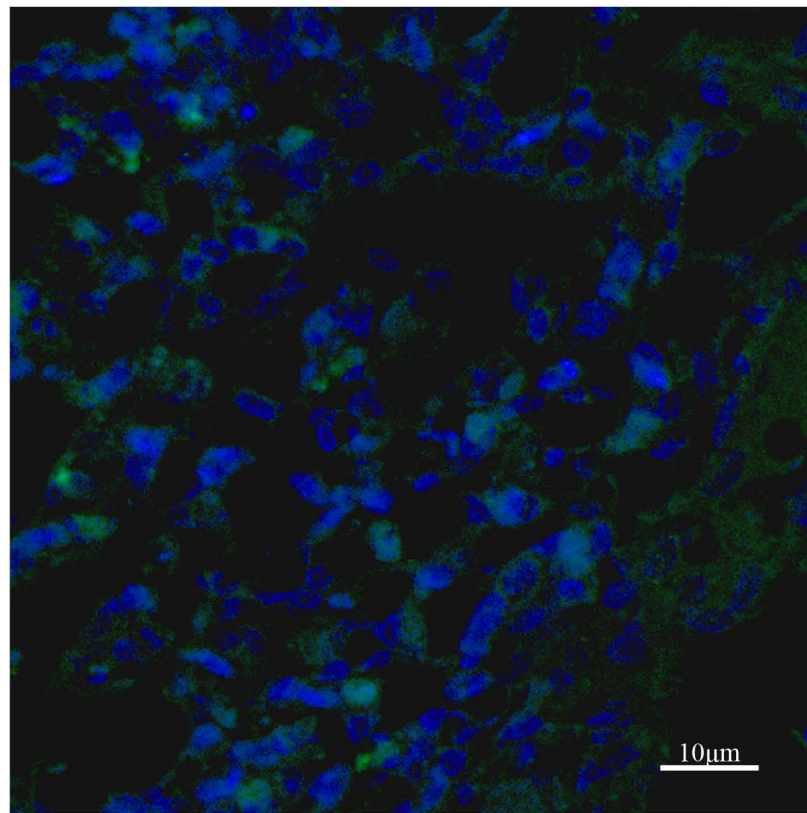

Heart

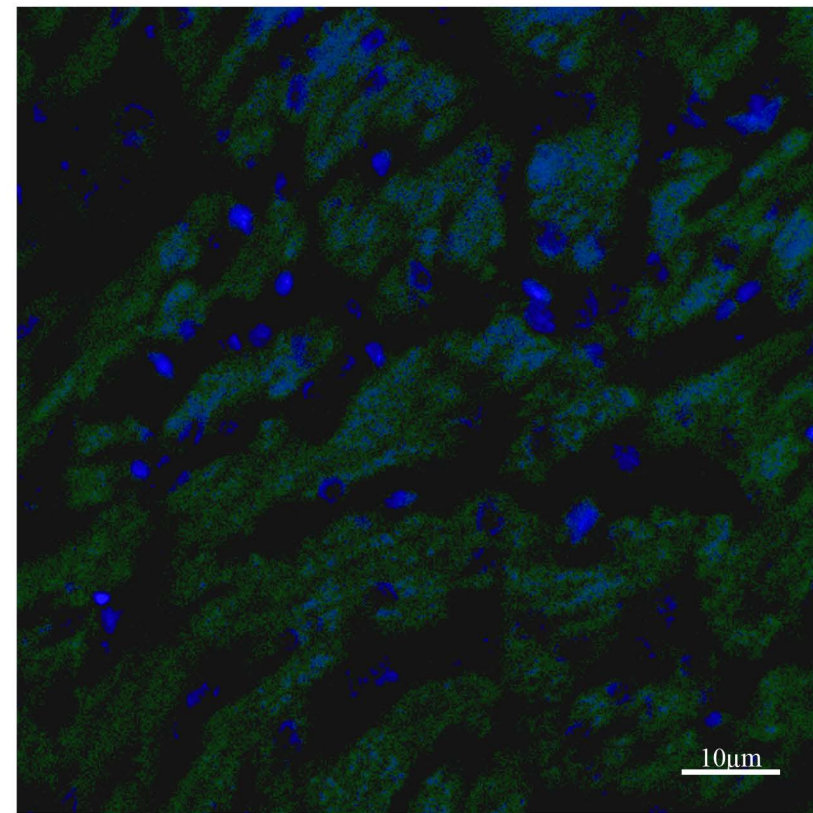

Supplement: Supplementary Materials — Immunofluorescence detection of viral antigen in inner organs of the chicken infected with HPAI A(H5N8) virus. Note: a: brain, b: lungs, and c: heart. Green: intracellular influenza A NP protein stained with anti-influenza A NP antibody (AA5H, Netherlands); blue: nuclei stained with 4′,6-diamidino-2-phenylindole (DAPI). Images were acquired by means of the LSM710/NLO microscope, using Plan-Apochromat 63x objective (Carl Zeiss, Germany). [file 4124865.f1.pdf]
